# Methodology of the Discrimination in the United States survey

**DOI:** 10.1111/1475-6773.13226

**Published:** 2019-10-27

**Authors:** John M. Benson, Eran N. Ben‐Porath, Logan S. Casey

**Affiliations:** ^1^ Department of Health Policy and Management Harvard T.H. Chan School of Public Health Boston Massachusetts; ^2^ SSRS Glen Mills Pennsylvania

**Keywords:** Determinants of Health/Population Health/Socioeconomic Causes of Health, Racial/Ethnic Differences in Health and Health Care, Survey Research and Questionnaire Design

## Abstract

**Objective:**

To describe survey methods used to examine reported experiences of discrimination against African Americans, Latinos, Asian Americans, Native Americans, women, and LGBTQ (lesbian, gay, bisexual, transgender, and queer) adults.

**Data Source and Study Design:**

Data came from a nationally representative, probability‐based telephone survey of 3453 US adults, conducted January‐April 2017.

**Methods:**

We examined the survey instrument, sampling design, and weighting of the survey, and present selected survey findings.

**Principal Findings:**

Examining reported discrimination experienced by multiple groups in a telephone survey requires attention to details of sampling and weighting. In health care settings, 32 percent of African Americans reported discrimination, as did 23 percent of Native Americans, 20 percent of Latinos, 18 percent of women, 16 percent of LGBTQ adults, and 13 percent of Asian Americans. Also, 51 percent of LGBTQ adults, 42 percent of African Americans, and 38 percent of Native Americans reported identity‐based violence against themselves or family members; 57 percent of African Americans and 41 percent of women reported discrimination in pay or promotions; 50 percent of African Americans, 29 percent of Native Americans, and 27 percent of Latinos reported being discriminated against in interactions with police.

**Conclusions:**

Even the small selection of results presented in this article as examples of survey measures show a pattern of substantial reported discrimination against all six groups studied.

## 1 INTRODUCTION

The purpose of the *Discrimination in the United States* survey, the results of which provide the basis for several articles in this special issue of *HSR*:* Health Services Research*, was to document the self‐reported prevalence of discrimination against six groups—African Americans, Latinos, Asian Americans, Native Americans, women, and LGBTQ (lesbian, gay, bisexual, transgender, and queer) adults—across multiple institutional and interpersonal domains. These domains include health services (health care) and social services (education, employment, housing, political participation, police, and the criminal justice system), as well as interpersonal domains that affect health outcomes, including slurs, microaggressions, harassment (sexual and nonsexual), and violence.

Data were obtained from a survey jointly designed by Harvard TH Chan School of Public Health, the Robert Wood Johnson Foundation, and National Public Radio and administered by SSRS, an independent research company. Interviews were conducted in English, Spanish, and Chinese, using random‐digit dialing, January 26‐April 9, 2017, among a nationally representative, probability‐based sample of 3,453 adults aged 18 or older. The sample included 802 African Americans, 803 Latinos, 500 Asian Americans, 342 Native Americans (defined as the Census category “American Indian or Alaska Native,” or AI/AN), and 902 white Americans, as well as 1596 women and 489 LGBTQ adults.

The survey and the articles in this issue bring a public health perspective to the complexity and pervasiveness of discrimination in the United States today. They attempt to examine discrimination by focusing on individuals' direct life experiences with discrimination, rather than on perceptions or beliefs about what US groups as a whole are generally experiencing discrimination. The survey differs from many prior studies in that it covers all six of these identity groups at the same time and asks them the same set of questions. This survey provides a wide‐angle lens of the experiences of multiple groups in America across multiple domains of life, all at the same point in time.

Because Harvard researchers were not directly involved in data collection and de‐identified datasets were used for analysis, the study was determined to be “not human subjects research” by the Harvard TH Chan School of Public Health Office of Human Research Administration.

## 2 METHODS

### 2.1 Survey instrument

Survey questions were developed after conducting a review of available questions on discrimination. This questionnaire was designed to ask the same series of questions on institutional and interpersonal discrimination across several separate groups, including African Americans, Latinos, Asian Americans, Native Americans, women, and LGBTQ adults, which was a methodological challenge requiring original question wording in order for question stems and response categories to work for all groups. The questionnaire was reviewed by external experts for bias, balance, and comprehension, and it was pretested in the field before it was conducted using the full sample. The complete survey instrument is shown in Appendix S1.

Discrimination was conceptualized as differential or unfair treatment of individuals based on self‐identified race/ethnicity, gender, or LGBTQ identity, whether that treatment is enacted by individuals (based on beliefs, words, and behavior) or social institutions (based on laws, policies, institutions, and related behavior of individuals who work in or control these laws, policies, or institution).^1^, ^2^, ^3^

The other articles in this issue analyze questions about personal experiences, covering six institutional and six interpersonal areas of discrimination. Institutional areas included were employment, education, health care, housing, political participation, and interactions with police and courts. Interpersonal areas included were racial/ethnic, gender, or anti‐LGBTQ slurs; microaggressions; other people's fear; sexual harassment; being threatened or nonsexually harassed; and experiencing violence. Also analyzed were two areas in which concerns about discrimination might prevent or deter adults from taking potentially needed action: seeking health and police services. We examined discrimination in domains previously demonstrated to be associated with health (eg, health care interactions),^4^, ^5^ as well as domains generally outside health services research (eg, police interactions), to capture a wide range of possible discriminatory experiences across respondents' lives. Questions about experiences were only asked among a random half‐sample of respondents to maximize the number of questions while limiting respondent burden. Questions were only asked of relevant subgroups (eg, college questions only asked among adults who had ever applied to or attended college). Questions on harassment, violence, and avoiding institutions for fear of discrimination were asked about yourself or family members because of the sensitive nature of the topics.^6^ Prior literature has demonstrated the validity of asking questions this way to measure experiences on sensitive topics, as vicarious experiences of stress (eg, through discrimination or harassment experienced by family members) can adversely affect the health of individuals, even without respondents directly experiencing it themselves.^7^

Screening questions regarding racial and ethnic identities were asked at the beginning of the survey. This method of screening also allowed interviewers to use the appropriate language in survey questions to describe or refer to the respondent's own identity. For example, this allowed questions to be read as “Did you experience [form of discrimination] because you are Latino?” rather than “because of your race or ethnicity?” This makes it possible to ask otherwise‐identical questions of respondents of each group while still specifying their own group identity. In turn, this enables researchers to see results for each group being asked the same questions during exactly the same time period.

### 2.2 Sample design

Phone numbers used for this study were randomly generated from cell phone and landline telephone sample frames, with an overlapping frame design. This means that a respondent could theoretically be sampled from both the cell phone frame and the landline frame. The sample plan consisted of three basic components: (a) stratified main sample RDD, where every adult qualifies and the general adult population respondents are reached by random‐digit dialing (RDD) of cell phones or landlines; (b) stratified RDD oversample, where respondents are reached by RDD cell phone or landline, and interviewed only if they were members of one of the racial or ethnic groups at the focus of the study, or if they were members of the LGBTQ community; and (c) prescreened omnibus sample, meaning callbacks to telephone numbers where respondents who were previously interviewed on the SSRS weekly omnibus poll indicated they were members of one of these racial or ethnic groups or fell under various definitions of LGBTQ. Representativeness was addressed by weighting procedures described below.

The RDD oversample was stratified to efficiently reach the targeted racial or ethnic subpopulations. To do so, both the cell phone and landline frames were divided into five components: (a) High Latino/Hispanic; (b) High black; (c) High Asian; (d) High AI/AN; and (e) Else. For cell phone numbers, the basis for the stratification was the estimated incidence of the group in the area covered by the rate center tied to the phone number. Rate centers are identifiers that tie cell phone prefixes to county and are used here only to improve accuracy in sampling. Because some people move and keep their phones, the respondent's actual location is determined by self‐reported zip code. For landline phone numbers, the strata were defined by the incidence of each racial/ethnic group in the Census block group associated with the telephone exchange (determined by the first six digits of the number). Estimates for the population counts, by group, in each rate center or exchange, were generated from Marketing System Group's (MSG) Genesys database. Table 1 specifies the criteria by which each stratum was defined for the cell phone and landline strata. Adults of all racial/ethnic groups were interviewed in each of the high‐density areas.

**Table 1:** Definitions of cell phone and landline strata

Stratum	Cell phone	Landline
High Hispanic/Latino	Rate centers in areas with 40% or more Hispanic/Latino	Exchanges in areas with 40% or more Hispanic/Latino
High black	Rate centers in areas with 37.5% or more black	Exchanges centers in areas with 37.5% or more black
High Asian	Rate centers in areas with 25% or more Asian	Exchanges in areas with 25% or more Asian
High American Indian/Alaska Native	Rate centers in areas with 12% or more American Indian/Alaska Native	Exchanges in areas with 25% or more American Indian/Alaska Native
Else	All other rate centers	All other landline exchanges

If in the process of screening for any racial/ethnic group member, respondents reported being LGBTQ, they were included in the LGBTQ oversample. In addition, the LGBTQ oversample included adults with telephone numbers where the respondent on the omnibus polls had reported that they were gay, lesbian, or bisexual (or volunteered they were transgender), which is a standard demographic question on that series of polls and slightly different from the questions asked on the *Discrimination in the United States* survey. Immediately prior to the survey's field period, a question asking whether respondents identified as transgender, genderqueer, or gender nonconforming was added to the omnibus polls and used for screening purposes. All respondents were screened about LGBTQ status, regardless of whether they were prescreened from the omnibus polls. No data from the omnibus polls were included in the *Discrimination in the United States* survey. Screening from the omnibus polls was used only to increase the likelihood of reaching an LGBTQ respondent.

The questionnaire was translated into Spanish and Chinese, so respondents could choose to be interviewed in either of these languages, or switch between the languages according to their comfort level. Those who preferred being interviewed in Spanish (n = 255) or Chinese (n = 33) were interviewed by bilingual interviewers.

### 2.3 Field procedures

#### 2.3.1 Pretesting

Live pretest of the survey instrument was conducted prior to the field period with respondents from both listed‐landline and prescreened samples. Interviews were completed with respondents from each of the racial/ethnic groups under study, and in the process, different genders and sexual orientations. SSRS provided a detailed summary of pretest findings, which included feedback from the interviewers. The final draft of the questionnaire was revised on the basis of the pretest. Changes, mainly relatively small wording modifications, were made in order to improve respondent comprehension of questions and the flow of the survey instrument.

#### 2.3.2 Survey administration

The field period for this study was January 26 through April 9, 2017. All interviews were completed using a Computer‐Assisted Telephone Interview (CATI) system, which ensured that questions followed logical skip patterns and that complete dispositions of all call attempts were recorded.

#### 2.3.3 Screening

The screening process for the survey involved the following procedure.

Cell phone respondents were interviewed once they confirmed they were 18 or older. In households reached via landline, the procedure varied by the number of adults in the household. In single‐adult households, the respondent answering the phone was interviewed, once she or he established they were 18 or older. In two‐adult households, the CATI program randomly selected whether the adult on the phone would be interviewed or the other adult in the household. In households with three adults or more (as well as where the person answering the phone refused to disclose the number of adults in the household), the interviewer asked to speak with the adult male or female (randomly selected) who had had the most recent birthday. If the other person selected was unavailable, another adult of the same gender was selected.

If the sample was part of the oversample component (meaning those parts of the sample where respondents were to be screened out if they did not meet race/ethnicity or LGBTQ specifications), the selected respondent was asked about their racial/ethnic identity, sexual orientation, and gender identification. Respondents qualified if they belonged to one of the oversampled racial/ethnic groups (ie, Latino/Hispanic, black, Asian, or AI/AN) or self‐identified as a member of the LGBTQ community.

#### 2.3.4 Multirace and Latino/Hispanic respondent self‐identification

In some cases, respondents identified as being multiracial. When that happened, interviewers asked respondents with which race they identified most, and any following questions about racial/ethnic discrimination or experiences were based on this self‐identification.

The US Census asks a question about Latino or Hispanic heritage separately from the question about race. Latino/Hispanic is not considered a race, and a Latino can be of any race. Researchers often use Latino/Hispanic as one group and then define all or most other races as excluding those who call themselves Latino or Hispanic. For instance, reports by the National Health Interview Survey often use this approach.^8^ Our survey generally follows this course. Respondents who said they were Latino/Hispanic were asked questions about discrimination experienced because they were Latino. One exception was made for the survey. Respondents who identified as Latino or Hispanic and as AI/AN were asked with which group they identified more, and ensuing questions were determined by their response.

#### 2.3.5 Efforts to maximize survey response

In order to maximize survey response, up to seven follow‐up attempts were made to contact nonresponsive numbers (eg, no answer, busy, answering machine); each nonresponsive number was contacted multiple times, varying the times of day and the days of the week that callbacks were placed using a programmed differential call rule; respondents were offered the option of scheduling a callback at their convenience; specially trained interviewers contacted households where the initial call resulted in respondents hanging up the phone; respondents reached by cell phone were offered $5 if they requested compensation for their time. A total of 113 respondents received incentives.

### 2.4 Weighting procedures

Data in the survey were weighted to provide nationally representative and projectable estimates of the adult population 18 years of age and older both overall and for each of the racial/ethnic groups and LGBTQ adults. The weighting process took into account the disproportionate probabilities of household and respondent selection due to the sample design and different types of telephones used by respondents and their households, as well as the probability associated with the random selection of an individual household member.

For the purposes of weighting only, respondents who identified as multiracial were not grouped based on which group respondents said they most identified with, but rather were considered separately as multirace, consistent with Census approaches. Similarly, respondents who were both AI/AN and Latino/Hispanic were considered AI/AN, and thus, Latinos/Hispanics were matched to non‐AI/AN Hispanic Census distributions.

Each race‐defined group was weighted using the following steps.

1. *Probability of selection* (*total*). A phone number's probability of selection depends on the number of phone numbers selected out of the total sample frame. For each respondent whose household has a cell phone number, based on self‐report, this is calculated as total cell phone numbers dialed divided by total numbers in the cell phone frame. For respondents answering at least one landline number, this is calculated as total landline numbers divided by total numbers in the landline frame.

The probability of respondent selection within households is also taken into account. In households reached by landline, a single respondent is selected. Thus, the probability of selection within a household is inversely related to the number of adults in the household.

Total probability of selection is calculated as the phone number's probability of selection (by frame), and for landlines, this is divided by the number of adults in the household. To avoid extremely large or small weights, the maximum number of adults was capped at 3. The sample weights derived at this stage are calculated as the inverse of the combined probability of selection.

- *Correction for oversampling of racial/ethnic groups and strata*. In order to correct for oversampling of telephone exchanges known to have higher densities of the targeted racial/ethnic groups and the corresponding undersampling of exchanges known to have lower densities, each case was assigned a weight equal to the share of its stratum among eligible landline or cell phone numbers divided by the share of its stratum in the sample, per each of the five strata (High Latino/Hispanic, High black, High Asian, High AI/AN, Else) for the targeted race/ethnicity groups.
- *Recontact propensity correction*. This adjustment accounts for the potential bias associated with recontacting respondents. Prescreened sample respondents were recontacted on the basis of their participation in a previous survey (ie, the weekly omnibus) and were treated, for the purposes of weighting, as if they were respondents to a panel study. Using inverse probability weighting (IPW) or propensity weighting, characteristics of the respondents as measured in the initial study were used to model the respondents' propensity to respond to the recontact survey. None of the data from the original omnibus polls were included in the main survey.
- *LGBTQ adjustment*. To address the fact that respondents who reported being LGBTQ were oversampled, the following correction was added to the weighting procedure. Data from the preceding 3 months of the omnibus poll for four groups of respondents (Hispanic/Latino, non‐Hispanic white, non‐Hispanic black, non‐Hispanic any other race) were raked to their demographics based on Census data. Then, estimates were run for LGB (lesbian, gay, or bisexual) or T (transgender, genderqueer, or other gender than male or female) based on these cases.

In the *Discrimination in the United States* survey itself, these were used as a base‐weight adjustment, meaning the unweighted percentages of LGB and T in the raw, unweighted data for each of the four racial/ethnic groups described above were adjusted to the percentages from the omnibus polls, before raking to Census demographics. The purpose was to correct for oversampling while not assuming what the ultimate weighted distribution would be.

- *Poststratification weighting* (“*raking*”). With the base‐weight applied, the sample underwent the process of iterative proportional fitting (IPF), in which each race group was balanced to match the known adult‐population parameters based on the 2016 March Supplement of the US Census Bureau's Current Population Survey (CPS). CPS estimates were derived from data downloaded from IPUMS.^9^ This process of weighting was repeated until the root mean square error for the differences between the sample and the population parameters was 0 or near‐zero.

Particularly important for the analyses presented in other articles in this special issue, poststratification weighting was done within each of the racial/ethnic groups. The population parameters used for poststratification were age (18‐29; 30‐49; 50‐64; 65+), gender, Census region (Northeast, Midwest, South, West), education (less than high school, high school graduate, some college, 4‐year college or more),^9^ and phone usage (cell phone only).^10^ Additional racial/ethnic group‐specific parameters included Hispanic heritage (Mexican, Puerto Rican, other) and birthplace (USA/Puerto Rico vs. another country) for Latinos/Hispanics, Asian heritage (Chinese, Vietnamese, other) for Asians, and living on tribal lands for Native Americans.^11^

- *Weight truncation* (“*trimming*”). The raking methodology used in sample calibration for each race group had the weights converging to match the population benchmarks. As is often the case with weighting, the resulting weights inflated the variance in the data, as measured by the ratio of the highest weight to the lowest weight and by the design effect due to weighting.^12^ This is commonly addressed by weight trimming,^13^ a method in which extreme weights, on the low and high ends,^14^ are identified and the weight distribution is truncated to reduce error in the estimates stemming from the increase in variance.

The trimming method used for this study was based on the weight distribution whereby a “prespecified probability of occurrence” marked an extreme point in the distribution that was then selected as the cutoff points for truncation.^15^ As Henry and Valliant note, this is a pragmatic approach typical of studies, such as this, constrained by deadlines, that would typically work well on the survey as whole, but could have more meaningful effects on some domain estimates.^16^

For most race groups, the distribution cutoff points were the 5 percent upper and lower bounds of the weights, with the exception being Hispanics for which the 2.5 percent bound was used. The decision was made considering the impact of trimming on representativeness as established by the population benchmarks.

- *Adjustment for race oversampling*. The combined weights (for each race group) were adjusted so that each group's proportion matched their proportion of the US adult population. For example, non‐Hispanic blacks were about 23 percent of the sample; the final adjustment weighted this group down to 12 percent to reflect their known share of the population, based on the 2016 March Supplement of the US Census Bureau's CPS.^9^

## 3 RESULTS

In this section, we consider seven main measures of survey performance and outcome.

### 3.1 Characteristics of the survey sample, by group

Table 2 shows the sample characteristics, unweighted and weighted, for each of the main groups. For the five racial/ethnic groups, US Census benchmarks are shown. Women and LGBTQ adults were not benchmarked separately from the raking of the total sample.

**Table 2:** Characteristics of the survey sample, by group

	Unweighted (%)	Weighted (%)	Benchmark (%)^a^
Latino/Hispanic			
Age			
Age 18‐29	24	28	29
Age 30‐64	60	62	61
Age 65+	15	11	10
Gender			
Male	51	50	50
Female	49	50	50
Education			
High school or less	47	63	64
Some college	23	21	21
4‐y college+	29	15	15
Region			
Northeast	17	13	14
North central	7	8	9
South	33	34	38
West	37	37	39
Birthplace			
USA/Puerto Rico	57	49	50
Another country	43	51	50
Hispanic heritage			
Mexican	55	59	61
Puerto Rican	11	9	9
Other	34	32	30
African American (non‐Hispanic)			
Age			
Age 18‐29	19	26	25
Age 30‐64	56	59	60
Age 65+	24	15	15
Gender			
Male	46	46	46
Female	54	54	54
Education			
High school or less	38	50	51
Some college	30	29	28
4‐y college+	32	22	21
Region			
Northeast	17	17	16
North central	16	16	17
South	53	56	58
West	12	8	9
Asian American (non‐Hispanic)			
Age			
Age 18‐29	37	24	22
Age 30‐64	50	61	62
Age 65+	13	15	15
Gender			
Male	64	50	47
Female	36	50	53
Education			
High school or less	14	27	30
Some college	19	19	18
4‐y college+	66	54	53
Region			
Northeast	19	20	21
North central	15	10	12
South	22	19	23
West	43	45	45
Birthplace			
USA/Puerto Rico	30	26	23
Another country	70	74	77
Asian heritage			
Chinese (not Taiwan)	31	24	23
SE Asian	17	23	24
Asian Indian	28	27	22
Other	24	26	31
Native American (American Indian/Alaska Native)			
Age			
Age 18‐29	13	23	30
Age 30‐64	60	61	60
Age 65+	27	16	11
Gender			
Male	54	50	48
Female	46	50	52
Education			
High school or less	44	60	58
Some college	28	24	27
4‐y college+	26	15	15
Region			
Northeast	5	6	9
North central	25	16	13
South	32	35	31
West	34	39	47
Live on tribal lands	32	23	22^b^
White (non‐Hispanic)			
Age			
Age 18‐29	15	18	18
Age 30‐64	60	59	59
Age 65+	36	23	23
Gender			
Male	54	48	49
Female	46	52	51
Education			
High school or less	20	39	40
Some college	25	26	25
4‐y college+	55	34	35
Region			
Northeast	17	18	19
North central	21	25	26
South	32	35	35
West	27	18	20
Women			
Age			
Age 18‐29	17	17	NA^c^
Age 30‐64	58	61	
Age 65+	24	23	
Education			
High school or less	32	42	
Some college	27	26	
4‐y college+	41	32	
LGBTQ			
Age			
Age 18‐29	34	41	NA^c^
Age 30‐64	54	51	
Age 65+	12	8	
Education			
High school or less	24	42	
Some college	27	26	
4‐y college+	49	32	

a US Census Bureau's Current Population Survey (CPS), March 2016 Supplement. CPS estimates were derived from data downloaded from Flood S, King M, Rodgers R, Ruggles S, Warren JR. Integrated Public Use Microdata Series, Current Population Survey: Version 6.0 [dataset]. Minneapolis, MN: IPUMS, 2018. https://doi.org/10.18128/D030.V6.0. Accessed June 24, 2019.^9^

b US Department of Health and Human Services, Office of Minority Health. *Profile: American Indian/Alaska Native*. March 2018. https://minorityhealth.hhs.gov/omh/browse.aspx?lvl=3%26lvlxml:id=62. Accessed June 24, 2019.^11^

c Women and LGBTQ respondents were not benchmarked separately from the raking of the total sample.

### 3.2 Margins of sampling error

Table 3 shows the sample sizes and margins of sampling error, accounting for design effect, for the survey overall (±3.2 percentage points at the 95% confidence level) and for each of the groups analyzed.

**Table 3:** Number of interviews, design effect, and margin of sampling error (trimmed and untrimmed weights), by group

	Unweighted sample size (N)	Untrimmed weight		Trimmed weight	
		Design effect	Maximum margin of sampling error	Design effect	Maximum margin of sampling error
Total	3453	3.9	±3.3%	3.7	±3.2%
White^a^	902	2.3	±4.9%	2.1	±4.7%
Latino/Hispanic	803	1.8	±4.7%	1.7	±4.5%
African American^a^	802	1.5	±4.2%	1.4	±4.1%
Asian American^a^	500	2.0	±6.2%	1.7	±5.8%
Native American (American Indian/Alaskan Native)	342	2.6	±8.5%	2.2	±7.9%
Women	1596	3.7	±4.7%	3.5	±4.6%
LGBTQ (lesbian, gay, bisexual, transgender, and queer)^b^	489	2.1	±6.4%	2.2	±6.6%

a African American, Asian American, and white American respondents who also identified as Hispanic or Latino were included only in the Latino sample.

b LGBTQ also includes people who are genderqueer and gender nonconforming.

### 3.3 Effect of weighting/design effect

Weighting procedures increase the variance in the data, with larger weights causing greater variance. Complex survey designs and post‐data collection statistical adjustments increase variance estimates and, as a result, the error terms applied in statistical testing. The design effect for each group is shown in Table 3.

### 3.4 Effect of weight trimming

Whereas trimming the weights typically serves to reduce variance and to increase the effective N, which reduces sampling error calculated for the survey's point estimates, this may also introduce bias in the data as the weighted data no longer converge at the weighting benchmarks. For each of the racial‐ethnic groups studied, design effect was reduced along with sampling error (see Table 3). Thus, on average, the estimates for any of these groups have a smaller confidence interval, though this is not guaranteed to be the case for each specific point estimate. As for bias, comparing the demographic makeup and responses to key survey questions as observed with trimmed and untrimmed weights, the overwhelming majority of trimmed demographics were within 1 percent of the untrimmed demographics. Accordingly, nearly all substantive results to key questions, using the trimmed weights, were within 1 percent of the untrimmed ones, and none exceeded a 3 percent difference (Appendix S2). Thus, on a substantive level, trimming did not meaningfully affect the results on these measures.

### 3.5 Completion and response rates

Table 4 shows the completion and response rates for the overall sample, the racial/ethnic groups, and LGBTQ adults. Among respondents who answered initial demographic screening questions, the overall completion rate was 74 percent. The overall response rate for this survey was 10 percent, calculated based on the American Association for Public Opinion Research's RR3 formula.^17^ This calculation takes into account that for the prescreened part of the sample, the total response rate is the product of the response rate for recontacts multiplied by the response rate of the original omnibus poll.

**Table 4:** Completion rates and response rates, by group

	Completion rate^a^	Response rate^b^
Total	74%	10%
Racial/ethnic groups		
White^c^	79%	16%
Latino/Hispanic	69%	10%
African American^c^	72%	11%
Asian American^c^	76%	8%
Native American (American Indian/Alaskan Native)	73%	9%
LGBTQ (lesbian, gay, bisexual, transgender, and queer)^d^	72%	9%

a Among respondents who answered initial demographic screening questions.

b Calculated based on the American Association for Public Opinion Research's RR3 formula.^18^

c African American, Asian American, and white American respondents who also identified as Latinos or Latino were included only in the Latino sample.

d LGBTQ also includes people who are genderqueer and gender nonconforming.

### 3.6 Telephone coverage

Interviewing was conducted by both cell phone (68 percent) and landline (32 percent) in order to ensure coverage of adults who use only one type of telephone.

### 3.7 Survey results for selected key outcome measures

The survey was designed to look at the self‐reported experiences across a wide range of domains and among several groups simultaneously, using parallel question wordings. Other articles in this issue detail the widespread prevalence of reported discrimination.

Table 5 presents data for a few selected measures. In health care settings, 32 percent of African Americans reported discrimination, as did 23 percent of Native Americans, 20 percent of Latinos, 18 percent of women, 16 percent of LGBTQ adults, and 13 percent of Asian Americans (by reported discrimination, we mean because of that particular identity, not others the person may have). In addition, 22 percent of African Americans, 18 percent of LGBTQ adults, 17 percent of Latinos, and 15 percent of Native Americans reported that they or a family member avoided health care over concerns about possible discrimination.

**Table 5:** Reported discrimination (weighted percent), by race/ethnicity and among women and LGBTQ adults^a^

	Subject of discrimination^b^	Whites	Blacks	Latinos	Native Americans	Asians	Women	LGBTQ Adults
		Weighted percent of respondents						
		(N = 479)	(N = 418)	(N = 375)	(N = 167)	(N = 266)	(N = 827)	(N = 230)
Going to a doctor or health clinic	You	5	32	20	23	13	18	16
Avoided doctor or health care because of concerns of discrimination/poor treatment	You or family member	3	22	17	15	9	9	18

		(N = 414)	(N = 367)	(N = 386)	(N = 167)	(N = 205)	(N = 718)	(N = 245)
Being paid equally or considered for promotions^c^	You	13	57	32	33	25	41	22

		(N = 423)	(N = 384)	(N = 428)	(N = 175)	(N = 234)	(N = 769)	(N = 259)
Interacting with police	You	10	50	27	29	18	15	16
Experienced violence	You or family member	13	42	20	38	10	21	51

Abbreviation: LGBTQ, lesbian, gay, bisexual, transgender, and queer.

a All questions asked among a randomized half‐sample of respondents within each category. Don't know/refused responses included in the total.

b Questions about “you” are personal experiences only; questions about “you or family member” ask if items have happened to you or a family member because you or they are [respondent's own racial/ethnic/gender/LGBTQ identity].

c Pay/promotion question only asked among respondents who have ever been employed.

Sizable proportions of adults from several groups also reported experiencing racial, gender, or LGBTQ identity‐based violence against themselves or family members, including 51 percent of LGBTQ adults, 42 percent of African Americans, and 38 percent of Native Americans. At least one in five adults in each minority reported discrimination in being paid equally or being considered from promotions, including 57 percent of African Americans and 41 percent of women. Half of African Americans (50 percent) and more than one‐fourth of Native Americans (29 percent) and Latinos (27 percent) reported being discriminated against in interactions with police.

## 4 DISCUSSION

In order to assess the *Discrimination in the United States* survey's methodology, we refer here to a summary chapter by Graham Kalton on how to survey hard‐to‐sample populations, such as those that are the focus of our survey.^18^ Kalton places an emphasis on probability sampling method, which he states is “necessary to provide the security of valid statistical inference.” Whereas many surveys of hard‐to‐sample groups are conducted by nonprobability methods, ours used telephone RDD probability sampling. There are several other types of probability approaches, which we did not use because of cost (in‐person) or time constraints (mail). Other methods, such as a sequential multimode format, often yield higher response rates than our telephone RDD approach.

Conditions for survey‐designed inference include known selection probability, high coverage of the target population, high response rates, weighting, and operational feasibility. Kalton states that it is often not possible to satisfy all of these criteria and some compromises are often needed. Our survey generally meets four of these criteria. The main drawback involves the low response rate (see limitations below).

Some of the techniques for sampling hard‐to‐reach populations include large‐scale screening, use of a large host survey for screening, disproportionate stratification, and multiple frames. Each of these approaches was utilized in our survey.

Kalton notes that the efficiency of screening is increased by concentrating the sample in areas where the population is more prevalent. In our survey, oversamples included high‐density African American, Latino/Hispanic, Asian, and AI/AN areas (Table 1). In addition, we were able to take advantage of a large series of weekly RDD omnibus polls to pool telephone numbers for adults with specific characteristics.

The data were weighted to compensate for the effects of oversampling. In addition, the data, overall and for each specific racial/ethnic group, were weighted using benchmarks from the US Census.

The selected survey results in Table 5 show the widespread prevalence of reported discrimination experienced by minorities across a number of domains. Other articles in this issue discuss in greater breadth and detail the experiences of each group.

Evaluation of our survey and its results should be interpreted considering several limitations. First, the experiences are self‐reported and therefore subjective. However, previous research has shown that perceived, self‐reported discrimination is associated with worse health outcomes.^1^, ^2^, ^19^, ^20^ Second, we examine whether people have experienced various types of discrimination, without regard to timing or severity. This limits the ability to detect current levels of discrimination and instead focuses on lifetime experiences, though discriminatory experiences may have long‐run effects on behavior and health.^2^, ^21^, ^22^, ^23^, ^24^

Third, the survey's low response rate is a notable limitation. The response rate, while low, is within the typical range of response rates for telephone polling by prominent nongovernmental survey organizations, which a 2017 study reported as 9 percent on average.^25^ Studies have shown that such surveys, based on probability samples and weighted using US Census parameters, yield accurate estimates in most cases when compared with both objective measures and higher‐response surveys.^25^, ^26^, ^27^, ^28^ For instance, a recent study showed that the average difference on several measures between government estimates from high‐response rate surveys and a Pew Research Center poll with a response rate similar to our survey was 3 percentage points. These measures included employment status, household size, health insurance status, length of residence at current address, marital and parenthood status, smoking, and having a driver's license.^25^ However, it is important to note that it is still possible that some selection bias may remain that is related to the experiences being measured and it is not clear what the size of such bias would be needed to reverse the findings.

Fourth, weight trimming/truncating, which was used in this survey, often introduces bias and may worsen margins of sampling error. In general, trimming/truncating is advisable only when the need for large weights cannot be addressed via sampling design and when it improves margins of sampling error, as was the case for this survey.

Fifth, we did not examine respondents' experiences being multiracial. If the respondent gave more than one race/ethnicity, they were asked which one they identified with most. This decision was made for practical reasons, chiefly that we could only examine so many groups and that being multiracial can involve a variety of racial/ethnic combinations that yield different experiences. Additional research is needed to explore experiences unique to multiracial adults.

## 5 CONCLUSIONS

An article by David Williams in this issue, as well as other prior research, has shown that major patterns of racism, sexism, and other discrimination can significantly harm the health and well‐being of impacted populations and that self‐reported discrimination is associated with worse health outcomes.^1^, ^2^, ^3^, ^19^, ^20^, ^21^, ^22^, ^23^, ^24^, ^29^ The *Discrimination in the United States* survey and the articles based on it extend prior work in this area by focusing on people's reports of their own and their family members' direct life experiences, rather than general perceptions of discrimination on the country; and by bringing together simultaneously these reported experiences across six groups, most of them underrepresented in much of public opinion research due to their low incidence in the population.

Surveying these hard‐to‐sample populations involves challenges, but with attention to the many particular aspects of sampling (especially oversampling and screening), coverage, and weighting, and taking into account limitations, it is feasible to conduct such surveys using probability‐based RDD telephone sampling.

Even the small selection of results presented in this article as examples of survey measures show a pattern of substantial reported discrimination against all six groups. The other articles in this issue explore reported discrimination against each of these groups, adding greater breadth and detail.
