# Supplementary material for: Methodology of the Discrimination in the United States survey
Source: Health Serv Res. 2019 Oct 27;54(Suppl 2):1389–98. doi: 10.1111/1475-6773.13226 (PMC6864373; doi:10.1111/1475-6773.13226)
Supplement: Supplementary file 2 [file HESR-54-1389-s002.docx]

**APPENDIX S1: SURVEY INSTRUMENT**

**A. Screening questions for groups**

S2. Are you, yourself, of Hispanic or Latino background, such as Mexican, Puerto Rican, Cuban, or other Latin American background?

(Ask all) (If S2=1 insert item in parentheses)

S1. (And besides being Latino,) What race or races do you consider yourself to be? Please select one or more of the following categories.

(Read list – enter all that apply)

1 White

2 Black or African American

3 Asian

4 American Indian or Alaska Native

6 Native Hawaiian or Other Pacific Islander

5 Some other race

(Ask if respondent is both Hispanic and American Indian/Native Alaskan or if gave more than one race at S1)

(Show only codes entered in S1; show code 0 if S2=1)

S2a. With which do you identify more?

(Read list - enter only one)

0 Hispanic or Latino

1 White

2 Black or African American

3 Asian

4 American Indian or Alaska Native

6 Native Hawaiian or Other Pacific Islander

5 Some other race (specify)

S3. Do you consider yourself to be…?

(If R says they are both trans and something else on the list, please code both)

1 Heterosexual or Straight

2 Gay or Lesbian

3 Bisexual

4 Or a different identity I haven’t mentioned (please specify)

5 (DO NOT READ) Transgender

6 (DO NOT READ) Queer

9 (DO NOT READ) Refused

(Ask all except if S3=5)

(If S3=5; gen in code 1)

TG-01. Some people describe themselves as transgender when they experience a different gender identity from their sex at birth. For example, a person who was raised male, but who identifies as female. Some people who do not identify as either male or female might also call themselves transgender. Do you consider yourself to be transgender?

1 Yes, transgender

2 Yes, genderqueer or gender non-conforming

3 No

9 (DO NOT READ) Refused

(Ask if respondent is not transgender or refused trans question)

S4a. What is your gender?

1 Male

2 Female

6 Or a different identity I haven’t mentioned (please specify)

9 (DO NOT READ) Refused

(Ask if TG-01=1,2 or S4a=6 – Ask if transgender or genderqueer or not male or female at S4a)

(If S4a=6 only show codes 3,4,5,6,9)

S4b How would you describe your gender? Select all of the following that apply.

(Read entire list – enter all that apply)

1 Male

2 Female

3 Transgender male

4 Transgender female

5 Genderqueer or gender non-conforming

6 Or a different identity I haven’t mentioned (please specify)

9 (DO NOT READ) Refused

(Ask if more than one code was entered at S4b)

(Display only codes selected at S4b)

S4c. With which do you identify most? (Read list)

1 Male

2 Female

3 Transgender male

4 Transgender female

5 Genderqueer or gender non-conforming

6 Or a different identity I haven’t mentioned (please specify)

9 (DO NOT READ) Refused

**B. Main Questionnaire**

Most questions regarding race, ethnicity, and gender were asked only of in-group members: for example, African-Americans were asked their views about discrimination against African-Americans. Latinos were asked their views about discrimination against Latinos. Women were asked about discrimination against women. In addition to the questions regarding race, ethnicity, and gender, questions regarding sexual orientation and gender identity were only asked of self-identified members of the LGBTQ community.

**I. Introduction**

**(Asked of half-sample A)**

S5. Have you ever applied for a job?

S6. Have you ever been employed for pay?

**(Asked of half-sample B)**

S7. Have you ever applied for college or attended college for any amount of time?

S8. Have you ever tried to rent a room or apartment or to apply for a mortgage or buy a home?

**II. Discrimination**

Q1. Generally speaking, do you believe there is or is not discrimination against (Respondent’s own racial/ethnic identity) in America today?

**(Asked of respondents who believe discrimination against their own race or ethnicity exists in America today)**

Q2. When it comes to discrimination against (Respondent’s own racial/ethnic identity) in America today, which do you think is the bigger problem? Discrimination that is based in laws and government policies, or Discrimination that is based on the prejudice of individual people?

**(Asked of whites)**

Q3. Generally speaking, do you believe there is or is not discrimination against racial or ethnic minorities in America today?

**(Asked of whites who believe discrimination against racial or ethnic minorities exists in America today)**

Q4. When it comes to discrimination against racial or ethnic minorities in America today, which do you think is the bigger problem? Discrimination that is based in laws and government policies, or Discrimination that is based on the prejudice of individual people?

**(Asked of respondents who identify as male or female)**

Q5. Generally speaking, do you believe there is or is not discrimination against (men/women) in America today?

**(Asked of respondents who identify as male or female and believe discrimination exists against their own gender in America today)**

Q6. When it comes to discrimination against (men/women) in America today, which do you think is the bigger problem? ? Discrimination that is based in laws and government policies, or Discrimination that is based on the prejudice of individual people?

**(Asked of respondents who identify as LGBTQ)**

Q7. Generally speaking, do you believe there is or is not discrimination against lesbian, gay, and bisexual people in America today?

**(Asked of respondents who identify as LGBTQ)**

Q8. Generally speaking, do you believe there is or is not discrimination against transgender people in America today?

**(Asked of respondents who identify as LGBTQ and believe discrimination exists against LGBTQ people in America today, i.e., if “yes” to either Q7 or Q8)**

Q9. When it comes to discrimination against LGBTQ people in America today, which do you think is the bigger problem? Discrimination that is based in laws and government policies, or Discrimination that is based on the prejudice of individual people?

Now we’d like to ask you some questions more specifically about what goes on in the general area where you live, as well as your personal experiences. When we say “the area where you live,” we just mean the general neighborhood, town, or part of town where you live.

**(Asked of American Indians/Alaska Natives)**

D8a. Do you live on tribal lands such as a reservation, pueblo, or Alaska Native village?

**(Asked of all racial/ethnic groups except American Indians/Alaska Natives)**

Q10. How well do you feel that your local government represents the views of people like you? Very well, somewhat well, not too well, or not well at all?

**(Asked of American Indians/Alaska Natives who live on tribal lands)**

Q10a. How well do you feel that your local tribal government represents the views of people like you? Very well, somewhat well, not too well, or not well at all?

**(Asked of American Indians/Alaska Natives who do not live on tribal lands; n= 232)**

Q10b. How well do you feel that your local government represents the views of people like you? Very well, somewhat well, not too well, or not well at all?

**(Asked of all racial/ethnic groups except American Indians/Alaska Natives)**

Q11. How much can people like you affect what your local government does? Would you say a great deal, some, only a little, or not at all?

**(Asked of American Indians/Alaska Natives who live on tribal lands)**

Q11a. How much can people like you affect what your local tribal government does? Would you say a great deal, some, only a little, or not at all?

**(Asked of American Indians/Alaska Natives who do not live on tribal lands)**

Q11b. How much can people like you affect what your local government does? Would you say a great deal, some, only a little, or not at all?

Now I have some questions about issues that may be facing people where you live. For each of the following, please tell me how frequently you think this happens to people where you live, using the scale often, sometimes, rarely, or never. Then I’ll ask if you’ve ever **personally** experienced this, not just where you currently live.

**(Asked of half-sample A)**

Q12. How often, if ever, do you believe (Respondent’s own racial/ethnic identity) where you live experience discrimination when applying for jobs? Often, sometimes, rarely, or never?

Q13. (Ask if respondent ever applied for a job) What about you? Do you believe you have ever personally experienced discrimination because (of/you are) (Respondent’s own racial/ethnic identity) when applying for jobs?

Q14. How often, if ever, do you believe (Respondent’s own racial/ethnic identity) where you live experience discrimination when it comes to being paid equally or considered for promotions? Often, sometimes, rarely, or never?

Q15. (Ask if respondent has ever been employed) What about you? Do you believe you have ever personally experienced discrimination because (of/you are) (Respondent’s own racial/ethnic identity) when it comes to being paid equally or considered for promotions?

Q16. How often, if ever, do you believe (Respondent’s own racial/ethnic identity) where you live experience discrimination when interacting with police? Often, sometimes, rarely, or never?

Q17. What about you? Do you believe you have ever personally experienced discrimination because (of/you are) (Respondent’s own racial/ethnic identity) when interacting with police?

Q18. How often, if ever, do you believe (Respondent’s own racial/ethnic identity) where you live experience discrimination when trying to vote or participate in politics? Often, sometimes, rarely, or never?

Q19. What about you? Do you believe you have ever personally experienced discrimination because (of/you are) (Respondent’s own racial/ethnic identity) when trying to vote or participate in politics?

**(Asked of half-sample B)**

Q20. How often, if ever, do you believe (Respondent’s own racial/ethnic identity) where you live experience discrimination when going to a doctor or health clinic? Often, sometimes, rarely, or never?

Q21. What about you? Do you believe you have ever personally experienced discrimination because (of/you are) (Respondent’s own racial/ethnic identity) when going to a doctor or health clinic?

Q22. How often, if ever, do you believe (Respondent’s own racial/ethnic identity) where you live experience discrimination when applying to college or while at college? Often, sometimes, rarely, or never?

Q23. (Ask if respondent ever applied to or attended college) What about you? Do you believe you have ever personally experienced discrimination because (of/you are) (Respondent’s own racial/ethnic identity) when applying to college or while at college?

Q24. How often, if ever, do you believe (Respondent’s own racial/ethnic identity) where you live experience discrimination when trying to rent a room or apartment or buy a house? Often, sometimes, rarely, or never?

Q25. (Ask if respondent ever tried to rent or buy a place to live) What about you? Do you believe you have ever personally experienced discrimination because (of/you are) (Respondent’s own racial/ethnic identity) when trying to rent a room or apartment or buy a house?

Now I’d like to ask you the same questions about things you or people where you live have experienced, but this time thinking about gender.

**(Asked of half-sample A respondents who identify as male or female)**

Q26. How often, if ever, do you believe (men/women) where you live experience discrimination when applying for jobs? Often, sometimes, rarely, or never?

Q27. (Ask if respondent has ever applied for a job) What about you? Do you believe you have ever personally experienced discrimination because you are a (man/woman) when applying for jobs?

Q28. How often, if ever, do you believe (men/women) where you live experience discrimination when it comes to being paid equally or considered for promotions? Often, sometimes, rarely, or never?

Q29. (Ask if respondent has ever been employed) What about you? Do you believe you have ever personally experienced discrimination because you are a (man/woman) when it comes to being paid equally or considered for promotions?

Q30. How often, if ever, do you believe (men/women) where you live experience discrimination when interacting with police? Often, sometimes, rarely, or never?

Q31. What about you? Do you believe you have ever personally experienced discrimination because you are a (man/woman) when interacting with police?

Q32. How often, if ever, do you believe (men/women) where you live experience discrimination when trying to vote or participate in politics? Often, sometimes, rarely, or never?

Q33. What about you? Do you believe you have ever personally experienced discrimination because you are a (man/woman) when trying to vote or participate in politics?

**(Asked of half-sample B respondents who identify as male or female)**

Q34. How often, if ever, do you believe (men/women) where you live experience discrimination when going to a doctor or health clinic? Often, sometimes, rarely, or never?

Q35. What about you? Do you believe you have ever personally experienced discrimination because you are a (man/woman) when going to a doctor or health clinic?

Q36. How often, if ever, do you believe (men/women) where you live experience discrimination when applying to college or while at college? Often, sometimes, rarely, or never?

Q37. (Ask if respondent ever applied to or attended college) What about you? Do you believe you have ever personally experienced discrimination because you are a (man/woman) when applying to college or while at college?

Q38. How often, if ever, do you believe (men/women) where you live experience discrimination when trying to rent a room or apartment or buy a house? Often, sometimes, rarely, or never?

Q39. (Ask if respondent has ever tried to rent or buy a place to live) What about you? Do you believe you have ever personally experienced discrimination because you are a (man/woman) when trying to rent a room or apartment or buy a house?

Now one more time, I’d like to ask you the same questions about things you or people where you live have experienced, but this time thinking about people who are lesbian, gay, bisexual, transgender, queer, or part of the LGBTQ community. I’ll ask about gay, lesbian and bisexual people separately from transgender and gender nonconforming people. We’ll still use the same scale for answers: often, sometimes, rarely, or never for people where you live, and just yes or no for if it has happened to you. Then we’ll move on to different questions.

**(Asked of half-sample A respondents who identify as LGBTQ)**

Q40. How often, if ever, do you believe gay, lesbian, or bisexual people where you live experience discrimination when applying for jobs? Often, sometimes, rarely, or never?

Q41. What about transgender or gender non-conforming people where you live?

Q42a. (Ask if respondent identifies as transgender or gender non-conforming and has ever applied for a job) What about you? Do you believe you have ever personally experienced discrimination because you are (Respondent’s own transgender, genderqueer, or gender non-conforming identity) when applying for jobs?

Q42b. (Ask if respondent identifies as LGBQ and has ever applied for a job) Do you believe you have ever personally experienced discrimination because you are (Respondent’s own LGBQ identity) when applying for jobs?

Q43. How often, if ever, do you believe gay, lesbian, or bisexual people where you live experience discrimination when it comes to being paid equally or considered for promotions? Often, sometimes, rarely, or never?

Q44. What about transgender or gender non-conforming people where you live?

Q45a. (Ask if respondent identifies as transgender or gender non-conforming and has ever been employed) What about you? Do you believe you have ever personally experienced discrimination because you are (Respondent’s own transgender, genderqueer, or gender non-conforming identity) when it comes to being paid equally or considered for promotions?

Q45b. (Ask if respondent identifies as LGBQ and has ever been employed) Do you believe you have ever personally experienced discrimination because you are (Respondent’s own LGBQ identity) when it comes to being paid equally or considered for promotions?

Q46. How often, if ever, do you believe gay, lesbian, or bisexual people where you live experience discrimination when interacting with police? Often, sometimes, rarely, or never?

Q47. What about transgender or gender non-conforming people where you live?

Q48a. (Ask if respondent identifies as transgender or gender non-conforming) What about you? Do you believe you have ever personally experienced discrimination because you are (Respondent’s own transgender, genderqueer, or gender non-conforming identity) when interacting with police?

Q48b. (Ask if respondent identifies as LGBQ) Do you believe you have ever personally experienced discrimination because you are (Respondent’s own LBGQ identity) when interacting with police?

Q49. How often, if ever, do you believe gay, lesbian, or bisexual people where you live experience discrimination when trying to vote or participate in politics? Often, sometimes, rarely, or never?

Q50. What about transgender or gender non-conforming people where you live?

Q51a. (Ask if respondent identifies as transgender or gender non-conforming) What about you? Do you believe you have ever personally experienced discrimination because you are (Respondent’s own transgender, genderqueer, or gender non-conforming identity) when trying to vote or participate in politics?

Q51b. (Ask if respondent identifies as LGBQ) Do you believe you have ever personally experienced discrimination because you are (Respondent’s own LGBQ identity) when trying to vote or participate in politics?

**(Asked of half-sample B respondents who identify as LGBTQ)**

Q52. How often, if ever, do you believe gay, lesbian, or bisexual people where you live experience discrimination when going to a doctor or health clinic? Often, sometimes, rarely, or never?

Q53. What about transgender or gender non-conforming people where you live?

Q54a. (Ask if respondent identifies as transgender or gender non-conforming) What about you? Do you believe you have ever personally experienced discrimination because you are (Respondent’s own transgender, genderqueer, or gender non-conforming identity) when going to a doctor or health clinic?

Q54b. (Ask if respondent identifies as LGBQ) Do you believe you have ever personally experienced discrimination because you (Respondent’s own LGBQ identity) when going to a doctor or health clinic?

Q55. How often, if ever, do you believe gay, lesbian, or bisexual people where you live experience discrimination when applying to college or while at college? Often, sometimes, rarely, or never?

Q56. What about transgender or gender non-conforming people where you live?

Q57a. (Ask if respondent identifies as transgender or gender non-conforming and has ever applied to or attended college) What about you? Do you believe you have ever personally experienced discrimination because you are (Respondent’s own transgender, genderqueer, or gender non-conforming identity) when applying to college or while at college?

Q57b. (Ask if respondent identifies as LGBQ and has ever applied to or attended college) Do you believe you have ever personally experienced discrimination because you are (Respondent’s own LBGQ identity) when applying to college or while at college?

Q58. How often, if ever, do you believe gay, lesbian, or bisexual people where you live experience discrimination when trying to rent a room or apartment or buy a house? Often, sometimes, rarely, or never?

Q59. What about transgender or gender non-conforming people where you live?

Q60a (Ask if respondent identifies as transgender or gender non-conforming and have ever tried to rent or buy a place to live) Do you believe you have ever personally experienced discrimination because you are (Respondent’s own transgender, genderqueer, or gender non-conforming identity) when trying to rent a room or apartment or buy a house?

Q60b. (Ask if respondent identifies as LGBQ and have ever tried to rent or buy a place to live) Do you believe you have ever personally experienced discrimination because you are (Respondent’s own LBGQ identity) when trying to rent a room or apartment or buy a house?

**(Asked of half-sample B)**

Q61. Have you ever avoided going to a doctor or seeking health care for you or others in your family out of concern for the cost?

Q62a. Have you ever avoided going to a doctor or seeking health care for you or others in your family out of concern that you would be discriminated against or treated poorly because… (insert item)?

a. you or they are (Respondent’s own racial/ethnic identity)?

b. you or they are a (man/woman)?

c. (Ask if identify as LGBTQ) you or they are a part of the LGBTQ community?

**(Asked of half-sample B)**

Q63. In your day-to-day life, have any of the following things ever happened to you, or not?

a. Someone referred to you or a group you belong to using a slur or other negative word

b. Someone made negative assumptions or insensitive or offensive comments about you

c. People acted as if they were afraid of you

(Ask about each item if the respondent said yes) Do you believe this happened to you because of your race or ethnicity, your gender, [if LGBTQ, read: your sexual orientation or gender identity], or was it for some other reason? You can select multiple answers.

**III. Employment & Education**

Please tell me whether you agree or disagree with the following statements about employment opportunities and the workplace. For each of the following statements about employment opportunities and the workplace, please tell me if you strongly agree, somewhat agree, somewhat disagree, or strongly disagree:

**(Asked of half-sample A)**

Q65. (Respondent’s own racial/ethnic identity) where I live have fewer employment opportunities just because they are (Respondent’s own racial/ethnic identity)**.** Do you…?

Q66. (Men/Women) where I live have fewer employment opportunities just because they are (men/women). Do you…?

Q67. (Ask of respondents who identify as LGBTQ) LGBTQ people where I live have fewer employment opportunities just because they are part of the LGBTQ community. Do you…?

Q68. (Ask of half-sample A respondents who are African American, Asian Americans, or American Indians/Alaska Natives) (Respondent’s own racial/ethnic identity) where I live are paid less than white people for equal work, because they are (Respondent’s own racial/ethnic identity)**.** Do you…?

Q69. (Ask of half-sample A respondents who are white) White people where I live are paid less than racial or ethnic minorities for equal work, because they are white. Do you…?

Q70. (Ask of half-sample A respondents who identify as female) Women where I live are paid less than men for equal work, because they are women. Do you…?

Q71. (Ask of half-sample A respondents who identify as LGBTQ) LGBTQ people where I live are paid less than non-LGBTQ people for equal work, because of their sexuality or gender identity. Do you…?

Please tell me whether you agree or disagree with the following statements about education and schooling. For each of the following statements about education and schooling, please tell me if you strongly agree, somewhat agree, somewhat disagree, or strongly disagree:

Q72. (Ask of half-sample B respondents who are African Americans, Asian Americans, or American Indian/Alaska Native)) Because of the way the schools operate where I live, (Respondent’s own racial/ethnic identity) here don’t have the same chances to get a quality education as white children. Do you…?

Q73. (Ask of half-sample B respondents who are white) Because of the way the schools operate where I live, white children here don’t have the same chances to get a quality education as racial and ethnic minority children. Do you…?

Q74. (Ask of half-sample B respondents who identify as male or female) Because of the way the schools operate where I live, young (men/women) here don’t have the same chances to get a quality education as (women/men). Do you…?

Q75. (Ask of half-sample B respondents who identify as LGBTQ) Because of the way the schools operate where I live, LGBTQ children here don’t have the same chances to get a quality education as children who are not LGBTQ. Do you…?

Now I have a different type of question, but still about education.

**(Asked of half-sample B)**

Q76. When you were growing up, were you encouraged to apply to college, discouraged from applying for college, or was this never discussed?

Q77. (Ask if discouraged) Do you think you were discouraged from applying because…

a. You are (Respondent’s own racial/ethnic identity)?

b. You are (man/woman)?

c. (Ask if respondent identifies as LGBTQ) You are part of the LGBTQ community?

**IV. Community Environment**

Q78. People often describe some neighborhoods or areas as predominantly one group or another, such as a predominantly black or white neighborhood. Would you say that the area where you live is predominantly (Respondent’s own racial/ethnic identity), or not?

Q79. (Ask of respondents who identify as LGBTQ) Would you say that the area where you live is predominantly LGBTQ people, or not?

Q80. If you were describing the place where you live, would you say that it is mostly upper income, mostly middle income, or mostly lower income, or is this something you don’t have enough information about to say?

**(Asked of half-sample B)**

Q81. If you were describing the health and well-being of the place where you live, would you say that it is mostly excellent health, good health, only fair health, or poor health, or is this something you don’t have enough information about to say?

Q82. (Ask everyone) Have you or a family member who is also (Respondent’s own racial/ethnic identity) been told or felt as though you wouldn’t be welcome in a neighborhood, building, or housing development you were interested in because (of/you are) (Respondent’s own racial/ethnic identity)?

Q83. (Ask of respondents who identify as LGBTQ) Have you or a friend or family member who is also part of the LGBTQ community been told or felt as though you wouldn’t be welcome in a neighborhood, building, or housing development you were interested in because you are part of the LGBTQ community?

Q84. (Ask everyone) Have you ever thought about moving or relocating to another area because you experienced discrimination or unequal treatment where you were living?

(If Yes, ask:)

Have you thought about moving because of discrimination, but haven’t actually moved or have you actually moved or relocated because of discrimination?

**(Asked of half-sample B)**

Q85. Compared to other places to live, do you think the (insert item) where you live is BETTER, WORSE, or ABOUT THE SAME as other places to live?

a. Availability of grocery stores

b. Air quality

c. Quality of drinking water

d. Quality of available housing

e. Quality of available doctors or health care services

f. Quality of public schools

g. Availability of local employment opportunities

h. Amount of crime

i. Availability of parks, green spaces, and recreational areas

j. Availability of public transportation options

**V. Public Safety**

Now I’d like to ask you some questions about public safety. Some of these questions will ask about the police in the area where you live. Many news events over the past few years have highlighted some of the tensions between police and the communities they work in. We want you to think about the place where you live and your own personal experience, rather than events nationwide.

**(Asked of half-sample A respondents who are African American, Asian Americans, American Indians/Alaska Natives)**

Q86. Do you believe that police officers where you live are more likely to use unnecessary force on a person who is (Respondent’s own racial/ethnic identity), or are they just as likely to use unnecessary force on a white person given the same type of situation?

**(Asked of half-sample A respondents who are white)**

Q87. Do you believe that police officers where you live are more likely to use unnecessary force on a person who is a racial or ethnic minority, or are they just as likely to use unnecessary force on a white person given the same type of situation?

**(Asked of half-sample A)**

Q88. Have you ever avoided calling the police or other authority figures, even when in need, out of concern that you or others in your family would be discriminated against because… (insert item)?

a. you or they are (Respondent’s own racial/ethnic identity)?

b. you or they are a (man/woman)?

c. (Ask if respondent identifies as LGBTQ) you or they are part of the LGBTQ community?

**(Asked of half-sample A)**

Q89. Have you ever avoided doing things that you might normally do, such as using a car or public transportation, seeking medical care, or participating in political or social events, because you wanted to avoid possibly interacting with the police or government authority figures?

(If yes)

Q90. How often would you say you avoided doing things you might normally do, to avoid the police or government authority figures? Frequently, sometimes, or not often?

**(Asked of half-sample A)**

Now I’m going to ask you a few questions about things that may have ever happened to you. Some of these may be difficult to think or talk about, but please do your best. Your honest answers can really help.

Q91. Do you believe that you or someone in your family has (insert item) because you or they are (Respondent’s own racial/ethnic identity)?

1. Experienced sexual harassment
2. Been threatened or non-sexually harassed
3. Been unfairly stopped or treated by the police
4. Been unfairly treated by the courts
5. Experienced violence

**(Asked of half-sample A respondents who identify as male or female)**

Q92. Do you believe that you or someone in your family who is also a (male/female) has (insert item) because you or they are a (male/female)?

1. Experienced sexual harassment
2. Been threatened or non-sexually harassed
3. Been unfairly stopped or treated by the police
4. Been unfairly treated by the courts
5. Experienced violence

**(Asked of half-sample A respondents who identify as LGBTQ)**

Q93. Do you believe that you or a friend or family member who is also part of the LGBTQ community has (insert item) because you or they are part of the LGBTQ community?

1. Experienced sexual harassment
2. Been threatened or non-sexually harassed
3. Been unfairly stopped or treated by the police
4. Been unfairly treated by the courts
5. Experienced violence

**(Asked of half-sample A respondents who identify as LGBTQ)**

Q94. Have you or a friend or family member who is also part of the LGBTQ community ever been verbally harassed when entering or while using a bathroom, or been told or asked if you or they were using the wrong bathroom?

**(Asked of half-sample A)**

Q95. Do you believe that the police force in your area reflects the racial or ethnic background of the people living in your area, or are the police mostly of a different racial or ethnic background than the people living in your area?

Q96. In the past year, have you been personally contacted by representatives of a political party, candidate, community organization, or ballot issue encouraging you to vote or support their cause during an election? For example, someone knocking on your door or calling you on the phone?

Q97. Are you registered to vote at your current address, registered to vote somewhere else, or are you not registered to vote?

Q97. Are you registered to vote at your current address, registered to vote somewhere else, or are you not registered to vote?

Q98. (If registered to vote) Did you vote in the 2016 presidential election when Hillary Clinton ran against Donald Trump, did something prevent you from voting, or did you choose not to vote?

**VI. Health Demographics**

Q99. In general, how would you describe your own physical health – excellent, very good, good, fair, or poor?

Q100. In general, how would you describe your own mental health – excellent, very good, good, fair, or poor?

Q101. Does any disability keep you from participating fully in work, school, housework, or other activities?

Q102. Has a doctor or other health care professional ever told you that you have a chronic illness, such as heart disease, lung disease, cancer, diabetes, high blood pressure, asthma or a mental health condition, or haven’t they?

Q103. Do you receive regular care from the Veterans Administration?

Q104. (Ask of American Indians/Alaska Natives) Do you receive regular care from the Indian Health Service or tribal or urban Indian clinics?

Q105. Do you have a regular doctor or health care professional that provides most of your health care when you are sick or have a health concern, or do you not?

Q106. Where do you usually go when you are sick or when you need advice about your health? Is it a hospital emergency room, a clinic at a hospital, a neighborhood clinic or health center, a private doctor’s office, or do you have no usual place of care?

Q107. Are you, yourself, now covered by any form of health insurance or health plan? This would include any private insurance plan through your employer or that you purchased yourself, as well as a government program like Medicare or Medicaid.

Q107. Are you, yourself, now covered by any form of health insurance or health plan? This would include any private insurance plan through your employer or that you purchased yourself, as well as a government program like Medicare or Medicaid.

(If covered by health insurance)

Q108. Which of the following is your **main** source of health insurance coverage? Is it a plan through your employer, a plan through your spouse’s employer, a plan you purchased yourself either from an insurance company or a state or federal marketplace, are you covered by Medicare or Medicaid, a plan through your parents, or do you get your health insurance from somewhere else?

**VII. Demographics**

Age

D3. What is the last grade or class that you completed in school?

D4. Are you currently employed full-time, part-time, or not currently employed?

D5. (Ask of Latinos) Were you born in the United States, on the island of Puerto Rico, or in another country?

D6. (Ask of Latinos) How about you and your family’s heritage. Are you Mexican, Puerto Rican, Cuban, Dominican, or are you and your ancestors from another country?

D7. (Ask of Asian Americans) Were you born in the United States or in another country?

D8. (Ask of Asian Americans) Now I want to ask you about you and your family’s heritage. Are you Chinese, Filipino, Asian Indian, Japanese, Korean, Vietnamese, or are you and your ancestors from another country?

D10. (Ask of American Indians/Alaska Natives) Are you currently enrolled as a member with a Native Americans tribe?

D11. Are you currently married, living with a partner, divorced, separated, widowed or have you never been married?

D12/D12a/D12b Income

D15. In politics today, do you consider yourself a Republican, Democrat, an Independent, or what?

**Appendix S2: Effects of Weight Trimming**

Table 1. Weight trimming for selected demographics and dependent variables, African Americans and Latinos (in percent)

|  | African Americans | | | Latinos | | |
| --- | --- | --- | --- | --- | --- | --- |
| Groups | Un-weighted | With un-trimmed weight | With trimmed weight | Un- weighted | With un-trimmed weight | With trimmed weight |
| Demographics |  |  |  |  |  |  |
| Gender |  |  |  |  |  |  |
| Male | 46 | 46 | 46 | 51 | 50 | 50 |
| Female | 54 | 54 | 54 | 49 | 50 | 50 |
| Age |  |  |  |  |  |  |
| 18-29 | 19 | 26 | 26 | 24 | 28 | 28 |
| 30-49 | 28 | 34 | 33 | 35 | 42 | 42 |
| 50-64 | 29 | 25 | 26 | 26 | 20 | 20 |
| 65+ | 24 | 14 | 15 | 16 | 10 | 11 |
| Education |  |  |  |  |  |  |
| High school or less | 38 | 51 | 50 | 47 | 64 | 64 |
| Some college | 30 | 28 | 29 | 23 | 21 | 21 |
| College graduate | 32 | 21 | 22 | 29 | 14 | 15 |
| Region |  |  |  |  |  |  |
| Northeast | 18 | 17 | 17 | 18 | 14 | 14 |
| Midwest | 16 | 17 | 17 | 8 | 9 | 9 |
| South | 54 | 58 | 57 | 35 | 37 | 37 |
| West | 12 | 8 | 9 | 39 | 40 | 40 |
| Metro Status |  |  |  |  |  |  |
| Urban | 32 | 33 | 33 | 25 | 24 | 24 |
| Suburban | 55 | 54 | 54 | 64 | 63 | 63 |
| Rural | 13 | 13 | 13 | 11 | 13 | 13 |
| % saying they have been discriminated against in each of these areas because of their race/ethnicity |  |  |  |  |  |  |
| When being paid equally or considered for promotions | 58 | 57 | 57 | 32 | 33 | 32 |
| When interacting with police | 51 | 50 | 50 | 29 | 28 | 27 |
| When going to a doctor or health clinic | 32 | 32 | 32 | 20 | 20 | 20 |
| When seeking housing | 44 | 45 | 45 | 27 | 31 | 31 |
| % saying they or a family member have personally experienced violence because of their race/ethnicity | 38 | 43 | 42 | 20 | 21 | 20 |

Table 2. Weight trimming for selected demographics and dependent variables, Asian Americans and Native Americans (in percent)

|  | Asian Americans | | | Native Americans | | |
| --- | --- | --- | --- | --- | --- | --- |
| Groups | Un-weighted | With un-trimmed weight | With trimmed weight | Un-weighted | With un-trimmed weight | With trimmed weight |
| Demographics |  |  |  |  |  |  |
| Gender |  |  |  |  |  |  |
| Male | 36 | 53 | 50 | 54 | 50 | 50 |
| Female | 64 | 47 | 50 | 46 | 50 | 50 |
| Age |  |  |  |  |  |  |
| 18-29 | 37 | 23 | 24 | 13 | 23 | 23 |
| 30-49 | 28 | 40 | 39 | 28 | 34 | 33 |
| 50-64 | 22 | 22 | 22 | 33 | 27 | 28 |
| 65+ | 13 | 15 | 15 | 27 | 16 | 16 |
| Education |  |  |  |  |  |  |
| High school or less | 14 | 29 | 27 | 45 | 62 | 61 |
| Some college | 19 | 18 | 19 | 28 | 23 | 25 |
| College graduate | 66 | 53 | 54 | 27 | 14 | 15 |
| Region |  |  |  |  |  |  |
| Northeast | 20 | 21 | 21 | 6 | 5 | 6 |
| Midwest | 11 | 12 | 10 | 26 | 16 | 17 |
| South | 23 | 21 | 21 | 33 | 37 | 37 |
| West | 46 | 47 | 48 | 36 | 41 | 41 |
| Metro Status |  |  |  |  |  |  |
| Urban | 22 | 24 | 24 | 12 | 14 | 14 |
| Suburban | 74 | 69 | 70 | 33 | 38 | 40 |
| Rural | 5 | 7 | 6 | 55 | 48 | 47 |
| % saying they have been discriminated against in each of these areas because of their race/ethnicity |  |  |  |  |  |  |
| When being paid equally or considered for promotions | 27 | 25 | 25 | 32 | 34 | 33 |
| When interacting with police | 16 | 20 | 18 | 31 | 29 | 30 |
| When going to a doctor or health clinic | 10 | 16 | 13 | 32 | 23 | 23 |
| When seeking housing | 18 | 28 | 25 | 25 | 18 | 17 |
| % saying they or a family member have personally experienced violence because of their race/ethnicity | 9 | 10 | 10 | 37 | 37 | 38 |
